# Associations between Risk Factors in Late Adolescence and Problem Behaviors in Young Adulthood: A Six-Year Follow-Up of Substance Related and Behavioral Addictions in Swedish High School Seniors

**DOI:** 10.3390/ijerph182312766

**Published:** 2021-12-03

**Authors:** Claes Andersson, Anders Håkansson

**Affiliations:** 1 Department of Criminology, Faculty of Health and Society, Malmö University, SE-205 06 Malmö, Sweden; 2 Department of Clinical Sciences Lund, Psychiatry, Faculty of Medicine, Lund University, SE-221 85 Lund, Sweden; Anders_C.Hakansson@med.lu.se

**Keywords:** longitudinal, tobacco, alcohol, illicit drugs, gaming, gambling

## Abstract

Introduction: Risk factors of traditional substance use related problems in young adults are more well-known than for behavioral addictions such as gambling and gaming problems. The present study aims to provide knowledge about the longitudinal patters of substance use related and behavioral addictions in early adulthood. Methods: Using self-report surveys, substance-related, psychiatric, and demographic predictors were assessed in Swedish high school seniors and re-assessed six years later along with gambling and gaming problems, *n* = 800. Associations (Risk Ratios) between risk factors in late adolescence and problem behaviors in young adulthood were analyzed. Results: Tobacco use, illicit drug use, and hazardous drinking in young adulthood were associated with tobacco use, illicit drug use, alcohol use, conduct problems, and impaired impulse control in late adolescence. Gambling problems in young adulthood were only associated with heredity of alcohol problems, while gaming was not associated to any problem behavior in late adolescence. Conclusion: It is concluded that predictors for traditional substance-related addictions differ from predictors for behavioral addictions, and that this difference is more pronounced for gaming problems than for gambling problems.

## 1. Introduction

Recent years have seen an increased research focus on behavioral addictions, i.e., addictive disorders not involving a substance. Among these, gambling disorders has a long-standing history as a diagnostic construct both in the Diagnostic and Statistical Manual of Mental Disorders (DSM-5) [1] and in the diagnostic classification of the World Health Organization (ICD-11] [2], although in the most recent versions of these manuals, the gambling disorder was formally categorized among addictive disorders instead of being classified as an impulse control disorder. Gambling disorder is characterized by a pattern of gambling with symptoms such as difficulty stopping or cutting down on gambling, ‘chasing losses’ behavior of coming back to gambling to make up for recent losses, and continued gambling despite severe consequences, such as indebtedness, social problems, and mental health issues [3].

In addition, since the most recent classification of the World Health Organization (WHO) [2], gaming disorders, representing a persistent and maladaptive behavior related to video gaming and similar gaming portals, has been introduced as a diagnosis within the section of addictive disorders. Thus, although still a condition for further research in the earlier DSM-5 manual, gaming disorder is now fully recognized as an addictive disorder by the WHO.

Risk factors of substance use in young adults are more well-known than for behavioral addictions. These appear to include a family history of alcohol use disorders [4], a range of contextual factors [5], factors related to peer attitudes and peer substance use in the young [6], and several psychiatric disorders occurring early in life [7], including ADHD [8,9] and typically disorders involving externalizing symptoms rather than internalizing ones [5]. Likewise, different types of substance use are likely associated to one another in longitudinal follow-up studies; for example, nicotine use has been shown to predict problem drinking in young adults [10].

The research knowledge on behavioral addictions is less extensive, given the shorter history of these conditions in clinical and policy-making settings. Problem gambling is known to affect a varying but significant minority of the general population in different settings; with prevalence numbers for problem gambling ranging from close to 0 percent to around six percent of the adult population [11]. Risk factors of developing problem gambling, including the diagnostic construct of gambling disorder, include male gender and mental health disorder [3,11]. Among individuals with gambling disorders, including in the young, co-morbidity with other mental health disorders is common [12].

For problem gaming, prevalence estimates in the general population are very few [13]. As an example of prevalence estimates available, Wittek and co-workers in Norway described a prevalence of likely problem gaming in 7.3 percent and addictive gaming in further 1.4 percent in people defined as gamers [14]. A Dutch study revealed a three-percent prevalence of addictive gaming in school children in their early teens [15]. Risk factors of problem gaming are hitherto sparsely researched. Despite an observed co-existence of problem gaming and psychiatric symptomatology, the actual predictive value of such symptoms has not been conclusive [13,16]. In particular, core symptoms of ADHD have been highlighted as potential predictors of problem gaming in the young [17].

Additionally, while gambling and gaming disorder represent the two first behavioral addictions recognized, these may share some features, but may also differ with respect to risk factors and clinical correlates; in population survey research, problem gambling and problem gaming appear to be statistically associated [18], although clinical features appear to differ, such that, for example, individuals with problematic gaming may be significantly younger [19].

In terms of longitudinal studies of correlates and predictors of addictive disorders, behavioral addictions have been researched to a lesser extent than substance use disorders. Additionally, while gambling has been assessed in several larger studies on risk factors, such research knowledge is sparser for gaming. In addition, there is very little research documenting longitudinal risk factors of both problematic alcohol use, drug use, problem gambling and problem gaming, simultaneously and in parallel, such that predictors of more traditional substance-related conditions can be compared to those related to the behavioral addiction more recently addressed in clinical settings. Therefore, the knowledge about trajectories towards one specific type of addictive behavior, instead of another, is limited, and it is unclear whether newer behavioral addictions represent the same risk factor profiles as expected from more traditional substance addictions.

For this reason, the present study, based on a previous school survey in adolescents in Sweden, aimed to provide knowledge about the longitudinal patterns of developing problematic substance use or symptoms of behavioral addictions in early adulthood. Specifically, the present study aimed to assess, in a six-year follow-up, the diverse predictive roles of adolescent substance use, adolescent mental health, and gender, for the development of problematic alcohol use, drug use, problem gaming, and problem gambling, respectively. Given the literature describing higher rates of psychiatric comorbidity in behavioral addictions, and the specific focus on core symptoms of ADHD, the study specifically addressed impulse-related and externalizing conduct problems as potential predictors, along with depressive and anxiety symptoms.

## 2. Materials and Methods

### 2.1. Setting

The present study was carried out in Sweden. With respect to behavioral addictions, these conditions are relatively novel to prevention and treatment systems in the present setting. As late as in 2019 gambling disorder was formally included among addictive disorders, for which the health care and social services are formally responsible, such that formal treatment initiatives for this condition prior that have been few [20,21]. With respect to gaming disorder, recently recognized in the WHO diagnostic system internationally, this manual yet has not been adopted to local conditions, such that diagnostic and formal uptake of treatment for gaming is hitherto not systematically established. Prevalence figures of problem gambling and gambling disorder, respectively, have been reported to be approximately 1.5 percent and 0.5 percent, respectively [21,22], whereas prevalence figures for problem gaming are hitherto largely unknown. Typically, most individuals with problem gambling are male, although there are indications of a narrowing gender difference in recent years [22], with possible even comparably high rates of problem gambling in chance-based online gambling [23]. For gaming behaviors and problem gaming, this is far less documented in epidemiological or clinical research, although the degree of problem gaming appears to be associated with higher rates of problem gambling [18].

### 2.2. Participants

This is a six-year follow-up study of Swedish high school seniors originally assessed in 2011 for participation in an intervention study on alcohol use [24]. The original study was approved by the Regional Research Ethics Committee (REC; File number 2010/4) and the follow-up study was later planned and approved separately by the same REC (File number 2017/4), thus the follow-up was not specified in the original study. Participants consented separately to the two studies, and informed consent was given in Swedish language.

The original assessment was made in 18 high schools in 9 different municipalities across the Skåne region in the south of Sweden. The schools were chosen to represent the Skåne region’s distribution between urban and rural areas, and where the western part of the region is highly urbanized, while the eastern part refers to rural areas. Since the study was originally planned as an intervention study were no relevant socio-demographic variables were collected.

A total of 2359 seniors were invited and the final sample comprised 2171 (92%) participants who provided informed consent, contact information, and completed a paper and pencil assessment battery. Average age was 18.83 (SD = 0.53), and 1214 (56%) participants were female.

Six years later, and by using previously provided contact information, participants in the original study were invited to complete a follow-up assessment in 2017. Out of 2171 participants in the original sample, a total of 800 (37%) consented and completed an online survey. Average age was 24.05 (SD = 0.34), and 525 (66%) participants were female.

Non-response was higher in males compared to females (71% vs. 57%). In relation to the measures of interest in the current study (see below), non-response was associated with tobacco use (51% vs. 39%), hazardous drinking (53% vs. 46%), conduct problems (9.9 + 11.1 vs. 8.8 + 7.6), and impaired impulse control (10.1 + 4.4 vs. 9.3 + 4.3). Response was associated with symptoms of depression and anxiety (3.3 + 2.7 vs. 2.9 + 2.9) at the time of the follow-up assessment.

### 2.3. Procedures

In the original study, researchers met with students in large groups to describe the purpose of the study. Completed consent forms were collected, and students who did not want to participate were excused. As an incentive for attendance, regardless of whether they consented to participate, all students in attendance were given a cinema voucher worth 100 SEK or $10 USD.

For the current follow-up study, those who had consented to participate in the original study were approached again by using the same contact information that had been provided during the initial study. Those who could be reached were asked to visit a website for information about the follow-up study, consent form, and online-assessment. As an incentive for participation, a gift card worth 150 SEK or $15 USD were given after consent and survey completion.

### 2.4. Instruments

#### 2.4.1. Conduct Problems

At the initial assessment were frequency of participation in a range of delinquent behaviors within the last 12 months measured with a 23-item scale [25]. Examples of the questions are “Hit someone for something they said or did” and “Damaged school or work property on purpose”. Each item was answered on a 5-digit scale ranging from 0 (never) to 5 (above 10 times), resulting in a total score ranging between 0 and 115. In the original study [25], the coefficient alphas were 0.89 for males and 0.80 for females. In the current study, the alpha score was 0.82.

#### 2.4.2. Impulsivity

At the initial assessment were the 19 items of the impulsivity sensation seeking (ImpSS) subscale of the Zuckerman–Kuhlman Personality Questionnaire used [26]. These items assess a lack of planning and tendency to act impulsively without thinking. Examples of the statements presented in the questionnaire are “I like to have new and exciting experiences and sensations even if they are a little frightening” and “I’ll try anything once”. Each item was answered with a true (1) or false (0), resulting in a total score ranging between 0 and 29. In the two original studies [26], alpha scores were 0.77 and 0.82. In the current study, the alpha score was 0.79.

#### 2.4.3. Family History of Alcohol and Drug Use

At the initial assessment were participants asked to respond whether they, to their own knowledge, had any biological relatives that have, or have had, a significant drinking or drug problem, respectively, and that should or did lead to treatment. Relatives refer to parents, siblings, and grandmother, grandfather, aunts, and uncles on both mothers and father’s side. This way of questioning was developed by Marlatt and Miller as part of the Comprehensive Drinker Profile [27]. Responses indicating no family history of alcohol and drug problem were coded as a no (0) and responses indicating any history of alcohol and drug problem were coded as a yes (1).

#### 2.4.4. Symptoms of Depression and Anxiety

At both assessments, the 8-item version of the Symptom Check List, SCL-8D, measured frequency of depressive and anxiety symptoms in the past 3 months [28]. This is a brief version of the Hopkins Symptom Checklist [29] including four items measuring symptoms of depression and four items measuring anxiety. For each of the 8 items, participants responded no (0) or a yes (1) to whether they have experienced any of the given symptoms during the past week. The total scale ranges from 0 to 8, and the subscales for depression and anxiety ranges from 0 to 4. In the original study [28], the alpha score was 0.80 for the total scale. In the current study, the alpha score was 0.89.

#### 2.4.5. Tobacco and Illicit Drug Use

At both assessments, participants were asked to report use of tobacco products or any illicit drugs, including cannabis, and prescription drug use without a prescription. The previous 12 months were assessed with measures derived from Monitoring the Future (MTF) [30], and the European School Survey Project on Alcohol and Other Drugs (ESPAD) [31], and the Customary Drinking and Drug Use Record (CDDR) [32]. Responses were coded as either a no (0) or a yes (1).

#### 2.4.6. Alcohol Use

Both assessments used the 10-item Alcohol Use Disorders Identification Test (AUDIT) [33]. The first three items measure quantity and frequency of alcohol use (AUDIT-C), three items cover signs of alcohol dependence, and the last four items assess alcohol related harm. Responses on each question are given on a 0–4 points scale. Cut-offs for hazardous drinking is (8) and 6 (women) on the total scale, and 4 (men) and 3 (women) on the consumption subscale. In the Swedish validation study [34], the alpha score was 0.82. In the current study, the alpha score was 0.80 at both the initial assessment and the follow-up assessment.

#### 2.4.7. Gambling Problems

At the follow-up assessment the NORD DSM-IV Screen for Gambling Problems (NODS-CLiP) [35] were used to identify those with gambling problems. The instrument consists of three items measuring preoccupation, loss of control, and having lied about gambling. Each item is coded as either a no or a yes, with a cut-off point of answering yes to one or more questions, which were coded as either no (0) or yes (1).

#### 2.4.8. Gaming Problems

At the follow-up assessment the 7-item version of the Gaming Addiction Scale (GAS) was used to identify those with problem gaming [36]. Examples of the questions are “have there been periods when all you could think of was the moment that you could play a game” and “have you had arguments with others about the consequences of your gaming behavior”. Answers are given on a 5-digit scale ranging from never to very often. Responding ‘sometimes’ or above on at least four questions is considered problem gaming, which were coded as either no (0) or yes (1).

### 2.5. Statistical Analysis

Analyses were conducted using the statistical package R (R Core Team, R Foundation for Statistical Computing, Vienna, Austria). To determine whether data collected at the initial assessment differed significantly from data collected at follow-up, the Fisher’s exact test was used for categorical data and the student’s t-test was used for ordinal data. Relative Risk (RR) was used to quantify associations between categorical variables collected at the initial assessment and categorical outcome variables. Fisher’s exact test was used to identify significant associations between categorial variables. The Wilcoxon signed-rank test was used to identify significant associations between nominal and quantitative variables. Since no relevant sociodemographic variables were collected, were no adjustments made for possible confounders. All tests were two-sided. A *p*-value of 0.05 was considered statistically significant. Bonferroni correction was used when analyzing associations between risk factors at the initial assessment and follow-up.

## 3. Results

### 3.1. Problem Behaviors in Late Adolescence and Young Adulthood

Sample characteristics at the initial assessment in late adolescence are displayed in Table 1. Comparisons of problem behaviors in late adolescence and in young adulthood is displayed in Table 2. It was found that all alcohol use variables were significantly reduced between the senior high-school year and late adolescence.

### 3.2. Associations between Risk Factors and Problem Behaviors

Table 3 displays associations between categorical risk factors in late adolescence and problem behaviors in young adulthood, and Table 4 displays associations between ordinal risk factors in late adolescence and problem behaviors in young adulthood. Tobacco use, illicit drug use, and hazardous drinking in young adulthood show similar associations. All these outcomes were associated with tobacco use, illicit drug use, alcohol use, conduct problems, and impaired impulse control in late adolescence.

After correction for multiple tests, gambling problems in young adulthood were only associated with heredity of alcohol problems, while gaming problems in young adulthood were not associated to any problem behavior in late adolescence.

## 4. Discussion

The present study provides preliminary evidence that behavioral addictions, in particular problem gaming, may display another risk factor profile than traditional substance-related conditions. Few substance-related, psychiatric, and demographic predictors were associated with problem gaming, and importantly, the level of hazardous alcohol drinking was a predictor of all substance-related conditions and problem gambling, whereas it demonstrated the opposite association for problem gaming. This suggests that problem gaming may be the first addictive behavior not demonstrating a clear positive association to other addictive behaviors, and possibly even the opposite.

Young adulthood is a transitional period in the life course, roughly coinciding with completion of high school and extending into the mid-20s, and is characterized by prolonged exploration of identity and life goals [37,38]. Risk behaviors are common in young adulthood. Across the life span, use of alcohol and illicit drugs, as well as corresponding disorders, are greatest between 18 and 25 years of age [39]. Recent studies also find that both gambling problems [11] and gaming problems [13] are highly prevalent in young adults. The current study analyzes how well risk factors at high school predict risk behaviors in young adulthood.

There are few longitudinal studies aiming to establish predictors for gaming problems in young adulthood. A scoping review by Richard and coworkers [13] reports that commonly reported risk factors for gaming disorders are emotional dysregulation and negative self-esteem, with depressive symptoms, inattentive symptoms, and social isolation being reciprocally associated with gaming disorder. Of the nine studies included in the review, follow-up periods ranged between six months and five years. The study with the longest follow-up period was conducted by Marmet and co-workers [40] and assessed men from age 20 to 25 using the six-item screener version of the Adult ADHD Self-Report Scale [41]. Marmet et al. [40] reported that gaming disorder had a bidirectional association with ADHD, meaning that ADHD increased the risk for gambling disorder and that gambling disorder increased the risk for ADHD. The current study found no association between gaming problem and impulse-related and externalizing conduct problems assessed with the Zuckerman–Kuhlman Personality Questionnaire [26]. Impulsivity and externalizing conduct problems is a group of symptoms within ADHD, which do not seem to be related to gaming problems. The associations between gaming disorder and ADHD needs further investigation.

Gender was not a significant predictor of any of the substance-related outcomes or gambling, whereas problem gamblers were significantly more likely to be male. While results have to be interpreted with some caution, given the low number of individuals detected with gambling and gaming behaviors, the lack of a gender difference for gambling may seem somewhat surprising. Problem gambling is traditionally associated with male gender, although the previously large gender difference [20,21] here may have started to narrow down in recent years [22], possibly due to the introduction of new gambling formats in the online setting [23]. However, in contrast, it is of no surprise that male gender predicted the measure of problem gaming [14,18]. Here, with the increase in online behaviors and thereby the availability of both gambling and gaming services to broader groups in the population, it cannot be excluded that gender patterns may start to change, and future research should assess this. Thus, where online gambling appears to narrow the prevalence gap in problem gambling between women and men, potentially, a similar trend could be seen for gaming in the future. Thus, gender issues remain of interest here, and should be monitored closely in larger study samples in the future.

The present findings add to the previous impression that individuals with problem gambling and problem gaming, representing the first two non-substance-related addictive disorders recognized, are likely to differ from one another [19]. The nature of video gaming and gambling for money, respectively, is diverse; gambling is a behavior where reward, or negative feedback, is immediate and can be repeated in a behavior corresponding to a loss of control behavior allowing for an increase beyond the extent and duration of gambling initially planned for by the individual. In contrast, video gaming typically does not include an immediate and distinct reward, but instead is favored by a continuous behavior of attempting to ‘level up’ within the game. In addition, age of onset in individuals with these problem behaviors are likely to differ [19]. Thus, in the present study, while respondents were in an age range where gaming typically occurs and may increase into an addictive behavior, problematic gambling behaviors may not fully have evolved in individuals in this age range. Additionally, importantly, women are typically older at the onset of problem gambling than their male counterparts [21]. Thus, for gambling, a survey study in young adults is less likely to capture problem gambling in women than in men.

Having said that gambling and gaming are inherently diverse phenomena [19], it should be borne in mind that a partial overlap in users and problem users may be seen. Importantly, the predominance of gambling in an online format in some settings may further contribute to partly close the gap between individuals involved in extensive gambling or gaming practices [18]. The latter may be further emphasized when actual gambling or gambling-inspired components are entered within the narrative of a video game, such that the user, within the video game, may encounter situations where real money of similar values are spent with the intention of winning in an immediate-reward hazard-based game. Thus, such in-game gambling may need to be assessed in further research, as it may put users at risk of both a problematic gaming and gambling behavior within the framework of video games.

The findings of the present study may have practical implications for clinical work in behavioral addictions, and for future research. In most settings, clinical work in the assessment and treatment of gaming disorder is unavailable or developed to a limited extent, and it is likely that such treatment is currently being developed in many settings. Although the present study results rely on a limited number of individuals with each of the non-substance-related outcomes, it shall be borne in mind in clinical work that the groups screened, diagnosed and treated for a gaming problem may not be the same as one would typically expect for other addictive disorders. In addition, based on the present preliminary results, it will be of utter importance to conduct further longitudinal studies in larger study samples, in order to further highlight the present findings. The fact that gambling disorder may have different risk factor profile than traditional substance use disorders, and that this is even more so for gaming disorder, needs to be confirmed in such future studies, in this and other geographical settings.

Strengths of this study include the longitudinal design, and the relatively large sample size of non-treatment seeking young adults. The main limitation to this study is that only 37% of the original population participated in the follow-up assessment, resulting in limited statistical power. It should also be considered as a limitation that no relevant sociodemographic variables were collected, which unfortunately limits the possibility to control results for confounding factors. The prevalence figures of the addictive behaviors assessed seem representative with figures ranging from 2 percent for gambling problems and 3 percent for gaming problems. Considering recent research on gaming problems [13,39], potentially important predictors including diagnostic criteria of ADHD were not assessed. There could be several reasons for the low response rates. At the initial assessment, contact information was collected by paper and pencil and could not be validated (e.g., written e-mail addresses could be false or illegible). Additionally, the years following secondary school tend to bring about major changes in both occupation and place of residence, and it seems likely that many have changed contact information during this period.

## 5. Conclusions

It is concluded that predictors for traditional substance-related addictions differ from predictors for behavioral addictions, and that this difference is more pronounced for gaming problems than for gambling problems. Problem gaming may be the first addictive behavior not demonstrating a clear positive association to other addictive behaviors, and possibly even the opposite.

## Figures and Tables

**Table 1:** Sample characteristics assessed in late adolescence (*n* = 800).

	Late Adolescence
Female	525 (66)
Heredity, Any	235 (29)
Heridity, Alcohol	213 (27)
Heridity, Drugs	44 (6)
Conduct problems	8.8 (7.6)
Impulse control	9.3 (4.3)
SCL-8D, Total	3.2 (2.7)
SCL-8D, Depression	1.8 (1.5)
SCL-8D, Anxiety	1.5 (1.4)

Note. Categorical (Gender and Heredity): Frequency (%). Ordinal (Conduct problems, Impulse control, SCL-8D): Mean (SD).

**Table 2:** Problem behaviors in late adolescence in comparison to young adulthood (*n* = 800).

	Late Adolescence	Young Adulthood	*p*
Tobacco use	308 (39)	272 (34)	
Drug use	112 (14)	89 (11)	
NODS-CLiP, Gambling problem	NA	15 (2)	NA
GAS, Gaming problem	NA	22 (3)	NA
AUDIT, Hazardous drinking	373 (47)	193 (24)	*
AUDIT-C, Hazardous drinking	531 (66)	434 (54)	*
AUDIT, Total	5.9 (4.7)	4.0 (0.1)	*
AUDIT, Consumption	4.1 (2.7)	3.3 (2.3)	*
AUDIT, Dependence	0.6 (1.3)	0.4 (0.8)	*
AUDIT, Harm	1.5 (2.4)	0.9 (1.8)	*

Note. Categorical (Tobacco use, Drug use, AUDIT/AUDIT-C: Hazardous drinking, Gambling problem, Gaming Problem): Frequency (%), Fisher’s Exact Test. Ordinal (AUDIT: Total, Consumption, Dependence, Harm): Mean (SD), Independent Samples T-test. NA = Not Available. * *p* < 0.05.

**Table 3:** Associations between categorical risk factors in late adolescence and problem behaviors in young adulthood (*n* = 800).

	Problem Behaviors in Young Adulthood				
	Tobacco Use	Hazardous Drinking	Drug Use	Gambling Problem	Gaming Problem
Female	0.8 [0.7, 1.0]	1.0 [0.7, 1.3]	0.8 [0.5, 1.1]	0.7 [0.2, 2.2]	0.3 [0.1, 0.8] *
Heredity, Any	1.3 [1.1, 1.6] *	1.1 [0.8, 1.4]	1.5 [1.0, 2.3]	8.3 [2.3, 30.5] **	1.1 [0.1, 2.7]
Heredity, Alcohol	1.4 [1.1, 1.6] *	1.1 [0.8, 1.5]	1.4 [0.9, 2.1]	9.1 [2.5, 33.3] **	1.2 [0.5, 3.0]
Heredity, Drugs	1.0 [0.7, 1.5]	1.0 [0.6, 1.8]	1.9 [1.0, 3.5]	1.5 [0.2, 11.6]	0.8 [0.1, 6.0]
Tobacco use	3.5 [2.8, 4.3] **	2.9 [2.1, 4.1] **	2.1 [1.4, 3.1] **	2.3 [0.7, 7.2]	0.5 [0.2, 1.4]
Drug use	1.9 [1.5, 2.3] **	1.6 [1.2, 2.1] *	2.8 [1.9, 4.3] **	1.2 [0.3, 5.5]	0.3 [0.0, 2.3]
AUDIT, Hazardous drinking	2.3 [1.9, 2.9] **	4.5 [2.8, 7.1] **	2.0 [1.3, 3.1] **	3.4 [0.9, 12.4]	0.5 [0.2, 1.2]
AUDIT-C, Hazardous drinking	2.8 [2.0, 3.8] **	2.8 [2.1, 3.7] **	2.0 [1.2, 3.3] *	5.4 [0.7, 41.3]	0.3 [0.1, 0.7] *

Note. Relative Risk (RR) [95% CI], Fisher’s Exact Test. * *p* < 0.05. ** Bonferroni adjustment.

**Table 4:** Associations between ordinal risk factors in late adolescence and problem behaviors in young adulthood (*n* = 800).

	Problem Behaviors in Young Adulthood				
	Tobacco Use	Hazardous Drinking	Drug Use	Gambling Problem	Gaming Problem
Conduct problems	3.0 [2.0, 4.0] **	4.0 [2.0, 5.0] **	3.0 [2.0, 4.0] **	5.0 [0.0, 10.0] *	1.0 [−1.0, 4.0]
Impulse control	2.0 [1.0, 4.0] **	2.0 [1.0, 2.0] **	2.0 [1.0, 3.0] **	2.0 [−1.0, 4.0]	−1.0 [−2.0, 1.0]
SCL-8D, Total	1.0 [0.0, 1.0] *	0.0 [0.0, 1.0] *	0.0 [0.0, 1.0]	0.0 [−1.0, 2.0]	0.0 [0.0, 2.0]
SCL-8D, Depression	0.0 [0.0, 0.0] *	0.0 [0.0, 1.0] *	0.0 [0.0, 1.0]	0.0 [−1.0, 1.0]	1.0 [0.0, 1.0]
SCL-D, Anxiety	0.0 [0.0, 1.0] *	0.0 [0.0, 0.0]	0.0 [0.0, 0.0]	0.0 [0.0, 1.0]	0.0 [0.0, 1.0]
AUDIT, Total	3.0 [3.0, 4.0] **	4.0 [3.0, 5.0] **	2.0 [1.0, 4.0] **	3.0 [0.0, 6.0] *	−2.0 [−5.0, 0.0] *
AUDIT, Consumption	2.0 [2.0, 2.0] **	2.0 [2.0, 3.0] **	1.0 [0.0, 2.0] **	2.0 [0.0, 3.0]	−2.0 [−3.0, 0.0] *
AUDIT, Dependence	0.0 [0.0, 0.0] **	0.0 [0.0, 0.0] **	0.0 [0.0, 0.0] **	0.0 [0.0, 0.0]	0.0 [0.0, 0.0]
AUDIT, Harm	1.0 [1.0, 1.0] **	1.0 [1.0, 1.0] **	1.0 [0.0, 1.0] **	1.0 [0.0, 2.0] *	0.0 [−1.0, 0.0]

Note. Median of difference [95% CI], Wilcoxon Rank Sum Test. * *p* < 0.05. ** Bonferroni adjustment.

## Data Availability

The data that support the findings of this study are available from the corresponding author, upon reasonable request.
